# Lived experiences of patients with distal renal tubular acidosis treated with ADV7103 and of their caregivers: a qualitative study

**DOI:** 10.1186/s13023-022-02294-w

**Published:** 2022-03-28

**Authors:** Michaël Acquadro, Alexia Marrel, Maria A. Manso-Silván, Catherine Guittet, Sophie Joukoff, Aurélia Bertholet-Thomas

**Affiliations:** 1 ICON Plc, Lyon, France; 2 Advicenne S.A., 21 Allée Boissy d’Anglas, 30000 Nîmes, France; 3 Centre de Référence des Maladies Rénales Rares–Néphrogones–Hôpital Femme Mère Enfant, Hospices Civils de Lyon–Filière ORKiD, Bron, France

**Keywords:** dRTA, Potassium citrate and potassium bicarbonate, Qualitative research, Patient-reported outcomes

## Abstract

**Background:**

Consequences of distal renal tubular acidosis (dRTA) on growth, bone and kidney, sometimes associated with hearing loss, may significantly affect quality of life (QoL). This descriptive qualitative study explores QoL linked to dRTA and gathers the impressions of patients with this rare disease (and caregivers) 5 years after enrolment in a clinical study, during which patients were treated with ADV7103, a prolonged-release granule formulation combining potassium citrate and potassium bicarbonate. Semi-structured, one-hour interviews with 6 adult and 13 paediatric patients with a confirmed diagnosis of dRTA and with parents of paediatric patients were performed using an interview guide. Qualitative analysis of anonymized interview transcripts based on grounded theory was conducted.

**Results:**

The main QoL domains impacted by dRTA and its treatment were education/work, social/family life, and emotional and physical well-being. ADV7103 (administered twice daily) was compared with the standard of care (SoC) taken before study entry (more than twice daily). Patients/parents reported that switching from previous SoC to ADV7103 had changed their lives:

- Difficulties at school due to burdensome administrative issues and need to explain disease and treatment affecting all families of paediatric patients (n = 13) disappeared, facilitating parents who had stopped working (to deal with their child’s treatment) to return to work,
- Family functioning was improved (n = 18), as travel and holidays became easier to organise and patients/parents stopped thinking about managing treatment daily/nightly, reducing tension in the family or couple,
- The emotional burden of disease perceived was relieved (n = 12) in the absence of treatment-related invasive questions from others,
- Gastro-intestinal adverse events and taste problems improved with ADV7103 (n = 18) and better compliance led to milder physical impacts and less need to be hospitalised.

The mean satisfaction score with ADV7103 compared to SoC was 9 out of 10 (10 = very satisfied). ADV7103 exceeded or met the expectations of 14 out of 17 patients that commented on that.

**Conclusions:**

Qualitative interviews show that dRTA and its treatment have a significant impact on QoL of patients and parents and that ADV7103 helps improve daily-life and reduces treatment burden, resulting in greater overall satisfaction of the patients and their families.

*Trial registration* EU Clinical Trials Register, EudraCT 2013-003828-36 on the 3rd of September 2013.

## Background

Distal renal tubular acidosis (dRTA) is a rare disease, due to impaired secretion of hydrogen ions at the distal tubule [1, 2], which can be of hereditary [3] or acquired origin [4, 5]. The disease is characterised by hyperchloremic metabolic acidosis in the presence of a normal plasma anion gap, and urinary pH > 5.5, resulting in renal calcium, phosphate and potassium wasting and hypocitraturia [6]. Clinical consequences of dRTA include failure to thrive/stunted growth, rickets/osteomalacia, bone fractures, nephrocalcinosis, nephrolithiasis [7]. If not adequately treated, dRTA causes abnormally low adult stature, damage to the bone and chronic renal disease. Additionally, there are genetic variants associated with deafness or late-onset deafness (e.g.,* ATP6V1B1* and *ATP6V0A4*) [7, 8].

The limitations of current alkalising standard of care (SoC) treatments for dRTA, such as dosing regimens requiring administration every three to six hours (sometimes also during the night), poor palatability and gastrointestinal tolerability, lead to adherence challenges [9] and render adequate metabolic control (i.e., normal plasma bicarbonate and urinary calcium excretion levels) challenging [10].

ADV7103 (Sibnayal) is a new alternative treatment for dRTA consisting of a combination of potassium citrate and potassium bicarbonate prolonged-release granules [11]. Comparatively, ADV7103 has the advantages of requiring only two doses per day (morning and evening), of being tasteless and easy to take, and of reducing gastrointestinal side effects, resulting in an adequate adherence to treatment and control of plasma bicarbonate and calciuria [12, 13].

Health regulatory authorities recommend assessing QoL through a patient-centred approach as a part of medical product development in order to incorporate patient experience data and other relevant information from patients and caregivers [14]. In the absence of specific quantitative tools to obtain patient reported outcome measures in the context of dRTA, qualitative research can be useful to document patient reported outcomes [15–17]. Due to the lack of responsiveness of existing generic patient reported outcomes and the challenge in developing specific patient reported outcomes questionnaires in rare diseases, qualitative interviews seem to be the most adapted strategy to measure patient experiences in patients with dRTA.

The aim of this descriptive qualitative study was to provide an insight into the impacts of dRTA, as well as to gather information about the lived experiences of the patients and their families with ADV7103 and with their previous SoC treatments.

## Methods

### Participants and setting

This study was conducted between June and November 2020 and involved qualitative in-depth interviews with patients with a confirmed diagnosis of dRTA and caregivers of paediatric patients. All patients included in a multicentre, single-arm, open-label, follow-up study (EudraCT 2013-003828-36) [13], proposed after a pivotal study (EudraCT 2013-002988-25) [12], and remaining in the follow-up study under treatment with ADV7103 at the time of the interviews, were invited to participate.

Patients and caregivers willing to participate provided informed consent to be interviewed. All participants were asked by the study investigators to complete a contact order form and were informed about their rights to access/modify their data in the study information sheet. The contact order form was sent by the investigators to the direct-to-patient contact team in charge of managing participants’ contact details securely. Each participant’s contact was then provided to the assigned interviewer for interview scheduling.

### Interviews

The interviewer was an independent male, PhD researcher in patient-centred outcomes, trained and experienced in qualitative interviewing. He did not have any relationship with the participants before the interviews, nor any particular personal interest in the research topic. Due to the nature of dRTA and its effect on hearing ability of some patients, an interpreter (parent/caregiver or professional interpreter) could be involved in the interview with the patients where applicable.

The interviews lasted approximately 1 h and were performed by telephone (except for one interview with an adult patient that was performed face-to-face at the hospital in the presence of a professional interpreter). In the case of paediatric patients, the first part of the interview took place with one of the parents, and the children were interviewed during the second part of the interview.

The data was collected using a semi-structured interview guide with open-ended questions. Non-directive interview techniques were used to avoid leading the discussion and to let the interviewees answer spontaneously. Probes were used to collect in-depth knowledge and information from participants. The themes discussed with patients and parents during the interviews included: diagnosis and symptoms of dRTA, impacts of the disease on QoL, impacts of treatment on QoL, motivations to participate in the clinical studies, and satisfaction with ADV7103.

The interviewer was instructed to ask the participants about their experiences prior to taking study product and how their experiences had changed since they joined the study and started taking ADV7103. If the participant did not spontaneously compare situations before and after ADV7103, the interviewer specifically probed the participants by asking about the time before the clinical study and the study period. Socio-demographic data were collected at the end of the interviews.

### Data analysis

All interviews were audio-recorded with the permission of the participants and uploaded to a secured platform. Audio-recordings were then transcribed verbatim by an external certified transcription agency and all the information that could identify a participant was removed from the transcripts. The transcripts were identified with the participant identification numbers used in the clinical study.

Thematic analysis based on grounded theory [18] was used for the analysis of the verbatim transcripts. Qualitative data analysis was performed by an iterative coding process driven by the objectives of the study and research questions [19, 20]. Segments of textual data were tagged with a code according to concepts discussed within the text. This code allowed to aggregate all text about the same concept across all interviews. Codes derived from the interview guide were used and new codes arising from the data were generated. Qualitative analysis was performed with MAXQDA (version 2020) software, a program designed to facilitate the organization, storage, coding, and retrieval of qualitative data using Boolean operators [21, 22].

Simple descriptive statistics were used to summarize socio-demographic data about the participants collected at the end of the interview.

### Saturation assessment

Sample size in qualitative research is guided by the principles of theoretical saturation. Saturation was assessed in order to provide a retrospective rationale for sample size. Saturation can be considered for a domain and a subdomain when a consistent pattern is reached in the responses of the participants and no new concepts emerge by conducting additional interviews [23, 24]. For each individual interview, in chronological order, the responses were analysed and compared to those obtained during the previous interview, in order to identify new concepts. A saturation graph, showing the cumulative percentages of concepts emerging after the successive interviews, was drawn.

## Results

### Participant demographics

Among the 27 patients remaining in the clinical study at the time of the interviews, 23 patients provided consent to be interviewed. Three of them could not be contacted and one refused the interview. A total of 19 patients were interviewed and included 6 adults (aged 19–27 years) and 13 paediatric patients (aged 6–17 years) with 12 parents (two of the patients were siblings), all French speaking. All patients had primary dRTA and were in the extension study for an average ± SD period of time of 62 ± 6 months. Patient and caregiver demographics are summarized in Table 1.

**Table 1:** Patient and parent/caregiver socio-demographic characteristics

	Adult patients (n = 6)	Paediatric patients (n = 13)	Parents/caregivers (n = 12)
Age (years), median (range)	24 (19–27)	10 (6–17)	44 (37–58)
Gender, female/male (n)	4/2	8/5	10/2
*Highest level of education (n)*			
Primary school	0	7	0
High school	0	6	6
Higher education (1st year; 2nd year)	5	N/A	3
Higher education (3rd year or more)	1	N/A	3
*Professional situation (n)*			
Full-time or part-time employment	2	N/A	8
Self employed	0	N/A	1
Student	3	13	0
Home	0	N/A	2
Searching for employment	1	N/A	1

### Saturation

**Fig. 1:**
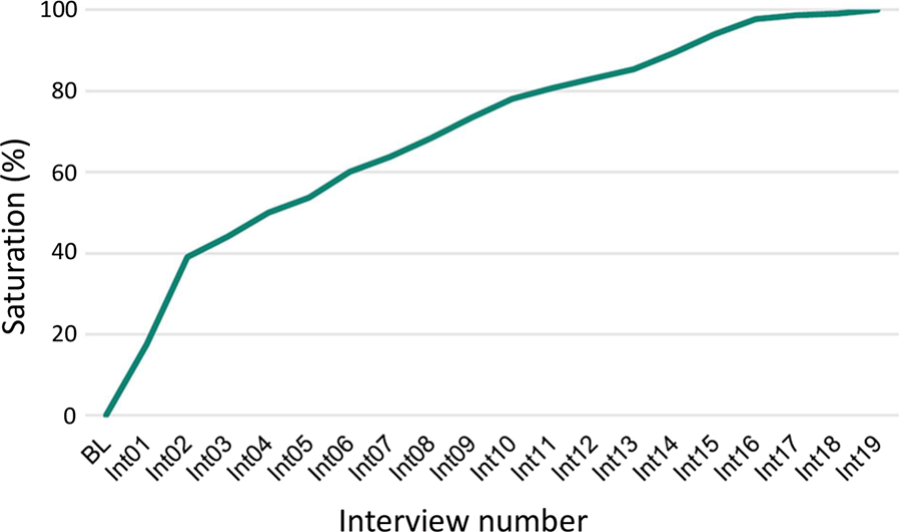
Saturation graph showing the cumulated percentage of new concepts

The saturation graph presented in Fig. 1 shows that saturation started to be reached at the sixteenth interview with the appearance of a plateau. From a total of 218 different codes, 40% of the concepts came from the first two interviews, 90% of the concepts were attained at the fourteenth interview, 98% from the sixteenth interview and 99% from the seventeenth interview. Two additional concepts emerged during the last interview.

### Diagnosis and symptoms

Patients experienced various symptoms that led to the initial diagnosis of dRTA. The symptoms mentioned were loss of weight (n = 7; 37%), tiredness (n = 6; 32%), gastro-intestinal (GI) disorders (n = 5; 26%), various renal problems (e.g., kidney stones, renal colic) (n = 5; 26%), hearing impairment (n = 4; 21%), failure to thrive (n = 3; 16%) and dehydration (n = 3; 16%). Patients who remembered when probed, indicated the diagnosis was performed at the hospital (n = 10; 53%), by a paediatrician (n = 3; 16%), or by a nephrologist (n = 3; 16%).

New symptoms reported to occur between diagnosis and start of the clinical trial were hearing impairment (n = 9, 47%) and tiredness (n = 6; 32%). Some participants reported an improvement in appetite (n = 4; 21%) and outlined gaining weight (n = 3; 16%) during this period.

The symptoms most frequently reported to occur during participation of the patients in the clinical trial were renal problems (n = 6; 32%) and tiredness (n = 5; 26%). Some patients reported improvement in appetite (n = 5; 26%) and less fatigue (n = 3; 16%) during this period.

### Impacts of the disease on QoL

The interviews indicated that the main QoL domains impacted by dRTA were education/work, social life, and emotional and physical well-being. The greatest impacts were in part attributed to hearing impairment: general difficulty with understanding others (n = 3, 16%), learning difficulties (n = 1, 5%), challenges at the workplace (difficulty in reading lips, n = 2; 11%, needing an implant, n = 1, 5%), speech impairment (n = 1, 5%), patient’s difficulty with trusting others because of their difference (n = 1, 5%).

Six patients (32%) reported that they had no problems talking about their disease with other people, while 3 patients (16%) said that they preferred not to talk about it. Some patients accepted their disease (n = 7; 37%). Some of them worried about the future (n = 6, 32%), and emotional consequences, on both children and their parents, such as depression (n = 3, 16%), anxiety related to the severity of the disease or to future availability of treatments (n = 2, 11%), and anger (n = 1; 5%), were mentioned during the interviews.

Two (11%) patients said that they felt too tired or weak to perform frequent physical activities and one patient (n = 1; 5%) indicated to have experienced pain at work related to kidney stones.

### Impacts of treatment

Patients reported using different SoC treatments before entering the clinical study and taking ADV7103 during the study, as summarised in Table 2. From the point of view of the patients at the time of the interview, treatment had a significant impact on their daily life. Comparisons of treatment experiences with SoC versus ADV7103 revealed several QoL domains that improved with the change of treatment (see Table 3 for illustrative quotes):

- *Difficulties at school/work* Difficulties at school/work with the previous SoC were reported in all the paediatric participants’ interviews (n = 13), due to burdensome explanations for school staff, burdensome administrative tasks, reluctance of school staff to provide treatment at school, and/or parents who stopped working due to repeated daily administration (at least 3 times a day). All of them (n = 13) found that with ADV7103 explanations for school staff were no longer needed, specific administrative tasks were no longer required, issues of reluctance to provide treatment at school were no longer present, facilitating a return to work for parents who had stopped working to take care of treatment organisation.
- *Social/family issues* Some of the participants, (31%, n = 6) reported difficulties with travel, holidays and daily-life due to treatment management during day/night, impaired ability of children to participate in social activities with their peers during lunchtime recess, and/or relationship difficulties between parents while taking SoC treatments. Most patients (95%, n = 18) found that with ADV7103 travel and holidays were easier to organise, they stopped thinking about managing treatment during day/night, resulting in reduced tension in the family or couple.
- *Emotional burden* Most of the participants (n = 13, 68%) felt uncomfortable with invasive questions from others in relation to their previous SoC medications, and 63% (n = 12) indicated that they felt relieved with ADV7103, leading to lower treatment burden, as they no longer needed to take their medication in front of others.
- *Physical burden* With the SoC, 84% of the participants (n = 16) reported bad taste, bad breath, or GI disorders. As a consequence, 21% of the participants (n = 4) refused to take the treatment and poor compliance leading to severe adverse events or strong physical impacts was reported by 37% of the participants (n = 7). With ADV7103, 95% of the participants (n = 18) reported neutral taste, and absence of bad breath and GI disorders. One of the participants (5%) refused to take the treatment on some occasions. Overall, better compliance was reported leading to less strong physical impacts (16% of the participants, n = 3).

**Table 2:** Characteristics of the treatments as self-reported by the patients during the interviews

	Previous SoC treatments	ADV7103
*Product*		
Sodium bicarbonate	8	0
Potassium citrate	7	19^a^
Missing data	4	0
*Form*		
Capsule	9	N/A
Powder	4	N/A
Syrup	7	N/A
Granule	0	19
*Number of daily intakes*		
2 ×	0^b^	18
3 ×	14	1^b^
4 ×	5	0
*Preparation*		
With water	8	15
Applesauce/yogurt	0	4
Missing data	11	0

^a^Corresponds to potassium citrate and potassium bicarbonate

^b^Not confirmed by clinical trial data

**Table 3:** Illustrative quotations about SoC and subsequent treatment with ADV7103 (translated from French original transcripts)

Concepts	Quotation examples (participant code number)
*Difficulties at school or work*	
Administrative burden	[About SoC] “And what was also complicated for him was that he ate in the canteen and therefore, since one of the treatments had to be taken at noon, we had to organise a PAI* with the school so that he could take his treatment and all that" (# 13)
Parents’ work	*[About SoC] “But at some point, that's why, like I said earlier, I quit my job. [About ADV7103] But now we have started normal life again. I went back to my job and all that, so did my husband. We have a normal life now, it's okay” (# 134)*
*Social/family issues*	
Difficulties travelling	*[About SoC] “During holidays, it was necessary to protect the capsules from heat. So, I remember episodes where, like when we were camping and we didn't have a fridge, we had to ask the campsite management to put the capsules in a cool place” (# 132)*
Social activities	*[About SoC] “During lunchtime, I had to leave for the infirmary and I couldn't meet, eat with my friends or whatever. No, they were going to eat and I was going to take my medicine” (# 35)*
Tension in the couple	*[About ADV7103] “This is a really great drug. It really helped me psychologically, it changed me, it changes a lot in life. [About SoC] Frankly, before, my husband and I, we would just argue. [About ADV7103] Since this drug came out, we are really, very, very well. I am very happy with this medicine" (# 134)*
*Emotional and physical burden*	
Invasive questions	*[About SoC] “…you can't see that he's sick and he didn't really want the friends to see it… Sometimes they would ask him: 'But, what are you taking?’ [About ADV7103] There it is. He was glad he didn't have to take treatment at lunch anymore so that he wasn't different from the others" (# 13)*
Adherence	*[About SoC] “Yes, sometimes I forgot and sometimes, I simply didn't feel like it, I didn't want to, so I skipped it. In fact, I wasn't really serious. Sometimes I would only take two doses […]. I was less consistent" (# 35)*
Taste	*[About SoC] "Too salty. She was having a hard time, she would swallow it anyway, but still, she didn't really like it. It was complicated, when she was younger […], [About ADV7103] but after the change of treatment, it went better" (# 16)*
Gastro-intestinal events	*[About ADV7103] “It's true that right from the start when she took it, there was no pain in her stomach at all. So that was a little bit of comfort for her" (# 121)* *[About ADV7103] “Fortunately, there was the last treatment… it has no effect on her digestion anymore so we are really happy” (# 72)* *[About ADV7103] “In fact, I was hungrier after the change of treatment" (# 11)*
*Satisfaction with ADV7103*	
Ease of use	*“Frankly, I have nothing to say, the truth. He takes it, I open the bag, hey, he opens his mouth, hey, he empties everything in his mouth. Afterwards he drinks water. Hop, everything goes down" (# 34/# 36)*
Ease of swallowing	*“So, they're little…like granules, little kernels. At first, she had a hard time swallowing them all, it was a bit complicated. Now it's going very well…She puts them all in her mouth and then she takes a big glass of water and she swallows them" (# 121)*
Sachet opening	*“It is not easy to open them [referring to ADV7103 sachets]. I don't always think about carrying around scissors or whatever, so sometimes it's annoying" (# 35)*
Dosage quantity	*"Frankly, nowadays it's okay, I take four sachets in the morning, four sachets in the evening, but at the very beginning, I took seven in the morning and seven in the evening. So, it was a lot harder and at the beginning I wanted to quit and all that, but afterwards well … Afterwards, they managed to diminish dosage little by little" (# 35)*
Efficiency	*“It is also more effective, over time. So, this is the goal, that the effects remain permanently to be able to help him, so this is a good step forward. We are happy regarding this” (# 72)*
Daily living	*“I find it much better than the one before, and I feel comfortable” (# 12)* *"It was a treatment that made our daily life easier anyway" (# 14)* *“The current treatment she has where she takes her dose in the morning and evening and then she's done with it. It’s life changing" (# 35)* *"Yeah, it's day and night compared to what I had before" (# 91)* *“Yeah, frankly, for us, it was a big, big relief to change medication. Frankly, it changed my child's life. As she says, it was very heavy to carry both physically and mentally" (# 121)* *"Yes, this treatment was a big change for us, a big springboard for better, I think, to support his disease" (# 121)* *“She's very happy, we as parents are the same. We can see the difference. We no longer recognize our child there who eats well, who finishes her plates. She asks for more to eat when she still wants more" (# 133)*

*PAI: *Projet d'accueil individualisé* (plan for personalized care at school)

### Motivations to participate in the clinical studies with ADV7103

When asked to indicate the reasons for participating in the pivotal clinical trial with ADV7103, 11 participants (58%) reported being motivated to reduce treatment burden for them or their children. A better treatment efficiency and to follow medical advice were second reasons to enter the clinical trial (n = 5 for both, 26%). Two participants (11%) were motivated to help other people find a new treatment, and one participant (5%) wanted to be followed by a specialist and an expert medical team.

When asked about reasons for remaining in the follow-up study, 2 participants (11%) reported they did not remember, and 2 participants (11%) did not answer the question. Eight participants (42%) said they were reassured about the effectiveness of the new treatment and/or that their child had accepted to continue. Six participants (32%) reported that they wanted to continue benefiting from the treatment. One participant (5%) said that he had met an efficient medical team that he wanted to keep, and one participant (5%) wanted to continue to contribute to science and medical research.

### Satisfaction with ADV7103

Overall, all patients reported treatment improvement with ADV7103 compared to SoC. They generally perceived the treatment as easy to use/take, efficient and without GI effects, and reported that it had made their lives easier (see Table 3 for illustrative quotes). When asked about it some participants suggested potential improvements to ADV7103, such as sachets easier to open, a lower number of granules to ingest, and a frequency of administration lower than twice daily. Others indicated that there was nothing to improve or that they just wished the product was available to everyone. In terms of treatment expectations, 4 patients (21%) reported that ADV7103 exceeded their expectations, 10 patients (53%) reported that it met their expectations, 3 patients (16%) indicated it was below their expectations, and two participants (11%) did not comment on this. When asked to rate satisfaction on a scale from 1 to 10 (1 = not satisfied to 10 = very satisfied), the mean score was 9, demonstrating a high overall satisfaction.

## Discussion

Directly interviewing patients and parents is critical to understand their lived experiences with the disease and its impact on their daily life and to understand changes brought by new treatments affecting their QoL.

The present qualitative research investigated the disease burden and treatment experience of 19 paediatric and adult patients with dRTA, who had been switched from previous SoC treatments to ADV7103 in the framework of a pivotal phase II/III clinical trial [12] and were followed up for at least 5 years during a phase III study [13]. This is the first time the impact of dRTA and its treatment on QoL, as described by the patients and their families, has been reported.

With the present approach, based on grounded theory [18], rather than applying a priori assumptions as in logico-deductive theory, the concepts emerge from the data, allowing the voice of the participants to be heard, and are then worked out in relation to the data throughout the duration of the research [20, 25, 26].

From the patient’s point of view at the time of the interview, SoC treatment had already improved some of the symptoms, reducing their impact. The greatest remaining impacts were related to the occurrence of deafness (not managed by alkalising therapy) and QoL impairments such as difficulties with studying (e.g., needing to go to a special school for the deaf and hearing impaired), social challenges (e.g., difficulties making friends), and negative emotional and physical impacts (e.g., depression, anxiety, weakness). Patients generally reported that they experienced few kidney-related symptoms and renal problems evoked by the patients were generally linked to the close kidney monitoring performed during the study rather than to actual occurrence of renal events.

However, the motivation of most patients/parents to participate in the first clinical study with ADV7103 indicated that the treatment they had before presented considerable limitations, as they were looking for less treatment burden and/or more efficient treatment. The interviews showed that current SoC treatments may present bad taste, tolerability issues, and require frequent dosing, which for diseases as dRTA, needing chronic treatment, could lead to poor medication adherence [27–29]. Simplifying treatment compared to current SoC, through reduction of the number of daily intakes and/or number of products required, as is the case with ADV7103 prolonged-release fixed-dose combination, may result in increased adherence to therapy and improved health outcomes [30–32]. The fact that the patients and/or their parents were reassured by both the effectiveness and acceptability of the product and that they wanted to continue to benefit from the treatment motivated most of the patients to pursue the follow-up study.

These interviews presented an opportunity to discuss with patients and caregivers with extensive, long-term experience with ADV7103, who indicated that the change in treatment had positive impacts on several aspects of their QoL which they considered as “life-changing”. The interviews stress the high level of satisfaction of the patients with the new treatment and, among the 17 patients commenting on that, 14 (82%) indicated that ADV7103 exceeded or met their expectations.

The main limitation of this study is that such a long period (more than five years) could have introduced a recall bias, when patients or their parents were asked about their past experiences. Also, interviews over the phone with deaf patients required an interpreter, and as parents often played this role to facilitate interviews with young children, this could have also led to some bias depending on the relationship between the child and the parents.

Despite these limitations, the qualitative interviews allowed to collect rich information about dRTA and its treatment as the patients and their parents kept good memory of SoC treatment satisfaction and experienced burden.

## Conclusion

In conclusion, this qualitative research study captures the perspectives from patients with dRTA and their parents, providing understanding of the challenges affecting the patients and their families on a daily basis. All participants were highly satisfied with the change of treatment from SoC to ADV7103, with benefits across multiple QoL dimensions. This treatment change was perceived as life-changing for patients/parents, supporting the use of ADV7103 as an alternative to SoC treatments.

## Data Availability

Data supporting the conclusions in this article are included within the article itself. Further data is available on request.
